# Advancing insights into virus-induced neurodevelopmental disorders through human brain organoid modelling

**DOI:** 10.1017/erm.2024.35

**Published:** 2024-11-26

**Authors:** Gabriella Crawford, Olivia Soper, Eunchai Kang, Daniel A. Berg

**Affiliations:** Institute of Medical Sciences, School of Medicine, Medical Sciences & Nutrition, University of Aberdeen, Foresterhill, Aberdeen, AB25 2ZD, UK

**Keywords:** 3D culture, brain development, disease modelling, human brain organoids, microcephaly, neurodevelopmental disorders, viral infection, induced pluripotent stem cells, iPSC, TORCH pathogens, vertical transmission

## Abstract

Human neurodevelopment is a complex process vulnerable to disruptions, particularly during the prenatal period. Maternal viral infections represent a significant environmental factor contributing to a spectrum of congenital defects with profound and enduring impacts on affected offspring. The advent of induced pluripotent stem cell (iPSC)-derived three-dimensional (3D) human brain organoids has revolutionised our ability to model prenatal viral infections and associated neurodevelopmental disorders. Notably, human brain organoids provide a distinct advantage over traditional animal models, whose brain structures and developmental processes differ markedly from those of humans. These organoids offer a sophisticated platform for investigating viral pathogenesis, infection mechanisms and potential therapeutic interventions, as demonstrated by their pivotal role during the 2016 Zika virus outbreak. This review critically examines the utilisation of brain organoids in elucidating the mechanisms of TORCH viral infections, their impact on human brain development and contribution to associated neurodevelopmental disorders.

## Introduction

Human brain development is a highly organised and complicated process, involving multiple stages of expansion during which many cell types play specific roles (Ref. 1). Species-specific differences in the developing brain between human and animal models, typically rodents, are apparent, particularly in terms of size, cytoarchitecture and cell types present (Refs 1, 2). For instance, rodent brains are smaller and exhibit smooth lissencephalic structures, whereas human brains are characterised by their larger size and complex cortical folding (Refs 2, 3). Additionally, structures such as the inner fibre layer and outer subventricular zone (oSVZ), containing intermediate progenitor cells (IPCs) and outer radial glial cells (oRGCs), are not present in developing rodent brains but play vital roles in human cortical expansion (Refs 1, 2). Proliferation and expansion of oRGCs within the oSVZ are associated with cortical neurogenesis and are a distinguished characteristic of gyrencephalic brain development (Ref. 2). Together, this highlights the importance of utilising and further developing humanised model systems that can more accurately recapitulate human neurodevelopmental processes.

Primary human brain tissue, sourced either from adult brains during surgical procedures or from electively terminated foetuses, offers the basis for alternative model systems for studying neurodevelopment and neurodevelopmental disorders in humans (Ref. 4). This tissue is particularly useful for modelling human-specific mechanisms and processes, as it contains all the essential cell types (Ref. 4). However, the research potential of primary tissue is limited as brain tissue can be difficult to access, may be difficult to culture long-term and often has an uncharacterised genetic background (Refs 4, 5).

In recent years, human brain organoids have emerged as valuable tools for modelling and studying various neurodevelopmental disorders. Brain organoids are self-assembling 3D cultures that exhibit functional and structural similarities to the foetal human brain (Refs 1, 6). They are generated from human stem cell-derived embryoid bodies cultivated under 3D growth conditions. Under such conditions, these stem cell aggregates possess the capacity to form organised structures, composed of neuronal progenitors, neurons and glial cell types (Refs 1, 6). Thus, recapitulating the cellular diversity, cytoarchitecture and developmental trajectory of the human brain. Human brain-specific features, such as gene expression patterns, prolonged neuroepithelium expansion, and enriched oRGC populations, are maintained in human brain organoids, making them valuable tools for studying complex developmental processes and modelling neurodevelopmental disorders (Ref. 6). Commonly referred to by various names—including cerebral, cortical and forebrain organoids—these terms are often used interchangeably. This review will adhere to the terminology of the original sources, using the more general term ‘brain organoid’ when appropriate.

Various environmental factors, including viral infections, have been identified as contributing to heightened risks of neurodevelopmental disorders, such as microcephaly, autism spectrum disorder (ASD) and schizophrenia (Refs 7, 8). During pregnancy, the placenta protects the foetus from many pathogens; however, vertical transmission can still occur (Refs 7, 9). Additionally, the exposure to viral infections can result in maternal immune activation (MIA), which in turn can be vertically transmitted to the developing foetus during any trimester (Refs 7, 10, 11). Various congenital neurological defects have been observed when viral infections occur during foetal development (Ref. 11). The mechanisms by which pathogens pass through the placental barrier and affect the developing foetus remain largely unknown, primarily due to the lack of accurate model systems that mimic human brain development and incorporate maternal factors (Refs 10, 12, 13). Therefore, the emergence of representative 3D organoid model systems has been critical for understanding how maternal viral infection and subsequent foetal viral infection impact human brain development (Ref. 10).

**Figure 1.**
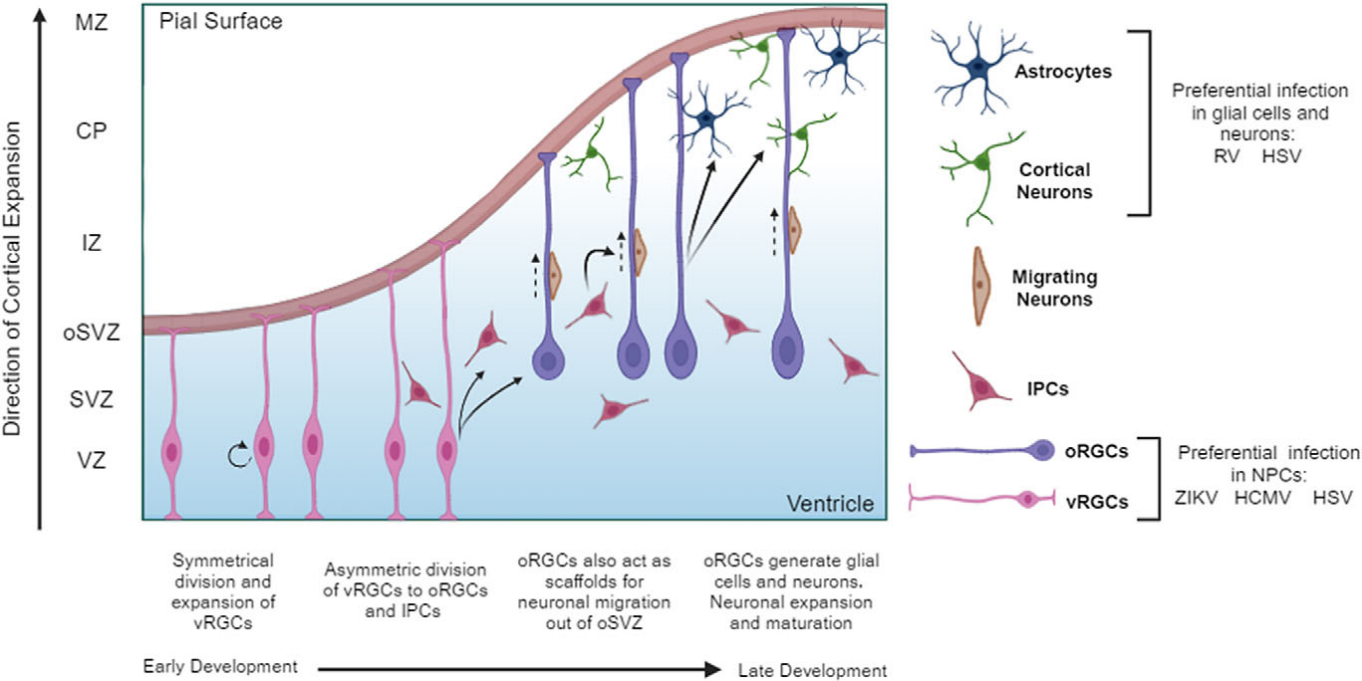
Schematic of cortical development and the impact of viral infection: This diagram illustrates the stages of cortical development and identifies the cellular processes and cell types most affected by viral infections. Initially, the cortex predominantly comprises neural stem cells (NSCs) and neural progenitor cells (NPCs), including ventral radial glia cells (vRGCs). During this early stage, NPCs are particularly vulnerable to infections from Zika virus (ZIKV), human cytomegalovirus (HCMV) and herpes simplex virus (HSV). As cortical development progresses and expands, vRGCs differentiate into intermediate progenitor cells (IPCs) and outer radial glia cells (oRGCs), which then evolve into more mature glial cells and neurons. In later stages of development, astrocytes and neurons become susceptible to rubella virus (RV) and HSV. The figure highlights how specific viral infections at different developmental stages lead to distinct effects on brain development and disease pathology. *Legend:* CP, Cortical plate; HCMV, Human cytomegalovirus; HSV, Herpes simplex virus; IPC, Intermediate progenitor cell; IZ, Intermediate zone; MZ, Marginal zone; NPC, Neural progenitor cell; NSC, Neural stem cell; oRGC, Outer radial glia cell; oSVZ, Outer subventricular zone; RV, Rubella virus; SVZ, Subventricular zone; vRGC, Ventral radial glia cell; VZ, Ventricular zone; ZIKV, Zika virus.

**Figure 2.**
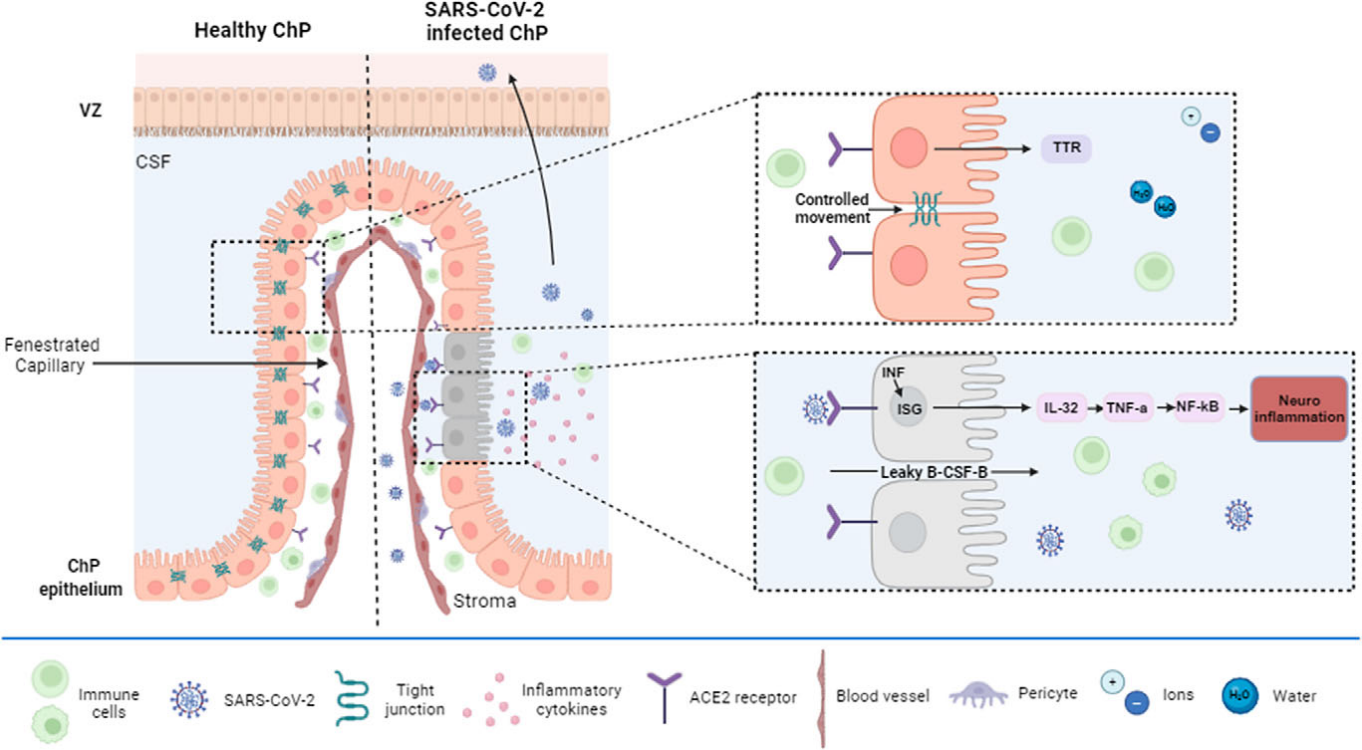
Interferon response in the choroid plexus following SARS-CoV-2 infection. A healthy choroid plexus (ChP), identified by markers such as transthyretin (TTR), maintains highly regulated tight junctions across the epithelial cell layer, controlling the movement of immune cells, ions, water and pathogens from the stroma to the cerebrospinal fluid (CSF). The ChP also secretes various growth factors and chemokines, which play a crucial role in proliferation, neurogenesis and development. Upon SARS-CoV-2 entering the ChP through the blood, viral particles pass through the fenestrated capillaries, bind to the ACE2 receptor and trigger an IFN-mediated immune response. IFNs activate interferon-stimulated genes (ISGs), leading to the production and excretion of cytokines and the induction of neuroinflammation. SARS-CoV-2 infection additionally leads to the downregulation of tight junction genes and breakdown of the B-CSF-B, allowing the dysregulated movement of immune cells, cytokines and viral particles to cross the ChP epithelium to the CSF, which in turn can enter the brain parenchyma. B-CSF-B, Blood-cerebrospinal fluid barrier; ChP, Choroid plexus; CSF, Cerebrospinal fluid; IL, Interleukin; IFN, Interferon; ISG, Interferon stimulated genes; TTR, Plasma transthyretin; VZ, Ventricular zone.

The TORCH acronym, first described in 1971 by A. Nahmias, referred to *Toxoplasma gondii*, rubella virus (RV), human cytomegalovirus (HCMV) and the herpes simplex viruses (HSVs) (type 1 and type 2) (Ref. 14). The ‘O’ was then altered to ‘Other’ to include more pathogens connected to prenatal infections, and further expanded to include syphilis, sometimes referred to as STORCH (Ref. 15). The classification and criteria of TORCH pathogens are continually discussed as more pathogens are linked to congenital defects and neurodevelopmental disorders (Refs 12, 13). Following the 2016 Zika virus (ZIKV) outbreak, ZIKV was added to TORCH, and more recently, studies have suggested including COVID-19, caused by severe acute respiratory syndrome coronavirus 2 (SARS-CoV-2) (Refs 12, 13, 15). TORCH pathogens have been reported to cause congenital defects such as heart defects, eye issues, pneumonia, brain calcifications and microcephaly, indicating that these pathogens can impact most major systems during foetal development (Ref. 16). They are also associated with intrauterine growth restriction, miscarriages and stillbirths (Ref. 15). Typically, all TORCH pathogens can infect women during pregnancy and are characterised by vertical transmission to the developing foetus, primarily through the placenta (transplacental) before delivery or via direct infection from the birth canal around delivery (Refs 12, 13, 15). This review will summarise studies using human brain organoids as a model system to investigate the impact of TORCH viral infections on brain development and the associated neurodevelopmental disorders, with a specific focus on viral pathogens, ZIKV, RV, HCMV, HSV-1 (Figure 1) and SARS-CoV-2 (Figure 2) (Table 1).

**Table 1.** Summary of 3D brain organoid models to study virus-induced neurodevelopmental disorders

Virus	Cellular phenotype	Modulated mechanisms	Therapeutics	Ref
ZIKV	Microcephaly-like growth restriction. Depletion of NPCs. Fewer and smaller VZ-like regions and lumen	Increased apoptosis and increased vRNA. Downregulation of cell cycle and cell division genes. Inhibition of IFN-mediated immune response genes, STAT2 degraded	Exogenous IFNβ – Increased VZ regions, rescued transcriptional changes, inhibited viral replication	(10)^a^
	Loss of organoid integrity	sfRNA induction of apoptosis. Downregulation of neuronal differentiation and neurodevelopment (DLX6/5, FOXg1). Induction of ISGs (OAS1/IFIT2) and pro-inflammatory cytokines (CXCL10). Inhibited Wnt-signalling, abnormal cell differentiation		(17)
	Microcephaly-like growth restriction. Thinning of VZ and loss of lumen. Infected NPCs	Impaired proliferation and increased cell death. Production of vsiRNAs in NPCs. Destruction of RNAi machinery	Enoxacin - Broad-spectrum antibiotic, RNAi enhancer, prevented infection and rescued microcephalic phenotype. Failed to prevent infection in Dicer KO organoids	(18)^a^
	Reduced organoid growth. Decrease in cell viability	Increase in cell death		(19)^a^
	Microcephaly-like growth restriction, reduction in cortical plate thickness and cortical neurons. Disruption to cortical organisation. Infected progenitor cells	Increase in TUNEL+/CC3+ apoptotic cells		(21)^a^
	Smaller size, loss of structure and integrity. Infection and reduction in NPCs	sfRNA induction of apoptosis/CC3+ cells. High levels of viral particles		(22)^a^
	Microcephaly-like growth restriction. Infected NPCs had rounded, unhealthy morphology and depleted NPC population	Activation of apoptosis, inhibition of shh and Ras–ERK signalling. Upregulation of TLR–3. Disrupted neuronal development. Downregulation of NTN1 and EPHB2	Thiophenecarboxamidopropionate compound. Competitively inhibited TLR3, rescued organoid size, NTN1 and EPHB2 expression and NPC death	(23)^a^
	Microcephaly-like growth restriction. Reduced VZ thickness. Infected NPCs and some infected IPCs, astrocytes and neurons	Decreased NPCs proliferation (EdU+). Increased activated caspase 3 apoptosis and cell death		(24)
	Reduction in organoid size. ZIKVE infection of RGCs	Increased apoptosis (CC3+ and ZIKVE+)	Cell entry factor AXL-inhibition - Not effective in rescuing organoid growth or cell death	(28)^a^
	Microcephaly-like growth restriction		SOF – Inhibited viral replication	(29)^a^
RV	Co-cultured with microglia cells which displayed high infection rates	Stimulation of IFN response and upregulation of IFI27/IFI6. Overall IFN response reduced but higher expression levels of IFITM3 in microglia-containing organoids. Dysregulated expression of genes for brain development (NFIB/NFIA). Decreased NOVA1 expression		(31)^a^
HCMV	Early-stage organoid growth unaffected			(10)^a^
	No large-scale cell death upon infection	Induced cell cycle dysregulation, upregulation of G2/M checkpoint genes and PI3/Akt/mTOR pathway genes. Upregulated IFN-α response. Downregulation of neurodevelopmental genes, including NES1, PAX6, SOX2/4, FEZF2, FOXG1, DMRTA2 and EMX1. Downregulation in cell–cell communication and ion-signalling (GJA1, CACNA1G, KCNF1)		(36)^a^
	Disrupted and unorganised organoid structure	Reduced calcium baseline and HCMV- cells responding to ATP stimulation. Reduction in neuronal differentiation (CTIP2+ and TUJ1+ cells)	MBV – Restored organoid structure and NPC differentiation to neurons. Increased ATP response in HCMV- cells	(37)
	Microcephaly-like growth restriction. Reduced SVZ and CP thickness. Impaired cortical layer formation. Infected fibroblasts and epithelial cells	Abnormal calcium signalling and neural network activity. Downregulation of ENO2, BNIP3, PDK1. Increased apoptosis and decreased proliferation. Overexpression of EGFR and PDGFRa. Downregulation of genes involved in neurodevelopment. Upregulation in immune and inflammatory response	Anti-PC specific NAbs, 1B2 and 62–11 inhibited infection and rescued organoid growth, cortical structure and SVZ/CP thickness	(38)
	Large vacuoles and cysts, filled with necrotic cells. Disrupted cortical structures, thin and elongated with abnormal cortical lamination. Viral IE1+ cells detected throughout organoid. Reduction in neuron populations			(40)^a^
HSV	Microcephaly-like growth restriction. Loss of integrity and disrupted cytoarchitecture. Impaired neuroepithelial identity. Fewer and smaller VZs, enlarged lumens with accumulating apoptotic cells. Chromatin marginalisation to nuclear periphery. Infected SOX2+ NPCs	Upregulated developmental, cellular processes and cell death pathways. Upregulated genes for proliferation, differentiation, apoptosis, transformation, AP–1 complex, NR4A3, NFIL3, DDIT3, ATF3, SNAI1. Lack of IFN-I and ISG induction. Viral protein ICP34. 5 restricts IFNβ activity	Exogenous INFα2 – Improved cytoarchitecture, suppressed infection, rescued NPCs. ICP34.5-null viral strain – reduced impact on organoid size and integrity, limited viral spread	(10)^a^
	Dysregulated cortical layers and brain organisation. Infected NSCs, impaired neural differentiation	Impaired neural differentiation, decrease in MAP2 and TUJ expression. Abnormal microglial and astrocyte activation, increase in IBA1+ and CD11b + cells and GFAP expression. Increase in inflammatory cytokines, TNF-α, IL–4/6/10		(42)^a^
	Reduced organoid growth. Disrupted organisation and integrity. Reduced length of neuronal processes and shortened axons	Disrupted neuronal processes	ACV – Not able to rescue organoid phenotype due to ACV resistance	(43)^a^
	Viral spread in organoid outer layers. Loss of tight junctions, cellular identity and organoid composition	Synaptic disfunction and dysregulation of SYN1, HOMER1 and STMN2. Reduced calcium activity and axonal outgrowth. Decrease in neuronal activity. Induced TNF-signalling pathway and increased p65 phosphorylation. Decreased proliferation	ACV – stopped viral replication, not able to rescue neuronal damage or TNF-signalling. ACV and NEC1/CDDO-Me co-treatment – Rescued TNF-pathway activation, rescued neuronal damage and neuroepithelial integrity	(44)
SARS-CoV–2	Using choroid plexus organoids, validated by TTR and AQP1 expression: ACE2 and TMPRSS2 are expressed on choroid plexus epithelial cells. Lipid-producing cells susceptible to infection. Loss of organoid integrity. Disrupted tight junctions and decrease in organoid internal fluid, breakdown of CSF-like barrier			(5)^a^
	Organoid expression of ACE2 and TMPRSS2	Upregulated viral entry factors FURIN, PLASMIN, CTSL1, NRP1, DPP4		(45)^a^
	Infected TUJ1+ and MAP2+ neurons	Infected neurons have disrupted tau distribution, tau hyperphosphorylation, neuronal cell death (TUNEL+)		(50)^a^
	Infects glial and choroid plexus cells. Co-localisation of SARS-CoV–2 NP and ACE2+ cells. Little infection of neurons or NPCs, with moderate infection of 5-HT2C+ choroid plexus cells and significant infection of GFAP+ and Nestin+ astrocytes and radial glial progenitor cells	Infected cells show apoptosis marker CC3, but few characteristics of cell death, such as DNA fragmentation		(51)
	Limited infection in region-specific cortical organoids, in neurons and astrocytes. Infection of TTR^+^ Choroid plexus organoids, with high levels of ACE2 expression	Infected ChP organoid cells formed syncytia, facilitated by Spike protein and ACE2. Increased cell death in infected and uninfected cells, TUNEL+. Upregulation in genes for inflammatory response, CCL7, IL–32, CCL2, IL–18, IL–8. Upregulated NF-kB and MAPK signalling pathways. Upregulated vascular remodelling genes NPPB and VCAN. Downregulation in genes for ion channels, transporters and cell junctions, AQP1, AQP4, SLC22A8. Downregulation of TTR. Increased inflammation and disrupted B-CSF-B		(52)^a^
	Infected Nestin+ NPCs and MAP2+ neurons. Infected CTIP2+, TBR2+, SATB2+ and CUX1+ neurons. Little MAP2+ cell death	Enriched pathways for viral entry, antigen presentation, negative neuronal projection development and the complement pathway. Increase in cell death, CC3+ and TUNEL+ cells. Decrease in excitatory synapses	SOF – 20 μM treatment inhibited SARS-CoV–2 replication and reduced cell death. Rescues excitatory synapses	(53)^a^
	Infected neurons, Spike+ and TUJ1+ cells. Increase neuron infection with enriched astrocyte populated organoids			(58)^a^
	Infected MAP2+ neuronal cells and SOX2+ NPCs. ACE2 expression in MAP2+ neurons	Increased neuronal cell death. Upregulated cell division, organelle fission and metabolic processes. Induced hypoxic state. Limited IFN and ISG response. Increased expression of genes for viral replication. SARS-CoV–2 negative cells had upregulated alcohol metabolism, cholesterol synthesis and cell death	Anti-ACE2 blocking antibody and patient-derived, CSF-containing antiviral antibodies - Inhibition of SARS-CoV–2 infection	(59)^a^
	Integrated pericytes into CP of organoid, healthy structure, astrocyte maturation and accumulation of laminin-β1, basement membrane. High infection of astrocytes	Upregulated neuronal differentiation, with fewer progenitors and more deep cortical layer neurons, upregulated GFAP, TBR1, DCX and STMN2 expression. Increased astrocytic cell death, CC3+ and p53+. Activation of IFN response in astrocytes, upregulation of IFIT1, IFI44, ISG15, STAT1, STAT2, USP18		(63)^a^

Brain organoids encompass cerebra, cortical and forebrain organoids. ACV, Acyclovir; AQP, Aquaporin; B-CSF-B, Blood-cerebrospinal fluid barrier; CC3, Cleaved caspase-3; ChP, Choroid plexus; CP, Cortical plate; CSF, Cerebrospinal fluid; HCMV, Human cytomegalovirus; HSV, Herpes simplex virus; IFN, Interferon; IL, Interleukin; IPC, Intermediate progenitor cell; KO, Knock out; NPC, Neural progenitor cell; NSC, Neural stem cell; PC, Pentamer complex; RGC, Radial glia cell; RNAi, RNA interference; RV, Rubella virus; sfRNA, Subgenomic flaviviral RNA; SOF, Sofosbuvir; TLR, Toll-like receptor; vsiRNA, Virus derived small interfering RNA; VZ, Ventricular zone; ZIKV, Zika virus; ZIKVE, Zika viral envelope protein.

a Studies also included neurospheres, mouse models, primary tissue and/or 2D cultures of stem cell-derived or primary cells to support or validate their organoid work.

## TORCH viral pathogens and their association with neurodevelopmental disorders

### Zika virus

ZIKV is a member of the *Flavivirus* genus, a group of mosquito-borne viruses, and is characterised by its enveloped structure with single-stranded RNA genome (Refs 17, 18). Vertical transmission of ZIKV has been evidenced by the presence of ZIKV in the placenta, amniotic fluid and blood of the developing foetus following maternal infection (Ref. 19). This foetal infection is associated with neurodevelopmental disorders including microcephaly and developmental delay, commonly known as congenital Zika syndrome, with more severe effects observed when exposed in early development (Refs 17, 18, 19). Human ZIKV strains were first identified in Africa in 1952 and Asia in 1969, but it was the public health emergency declared by the World Health Organization in 2016 that highlighted the increasing cases of ZIKV infection and associated microcephaly (Ref. 20). Since then, ZIKV has severely impacted Brazil and much of the Americas, with no approved vaccines or antiviral drugs available to treat or prevent ZIKV infection or microcephalic phenotypes (Refs 18, 21). Therefore, it is imperative to understand the mechanisms behind ZIKV infection, the viral transmission, its impact on developing foetuses, and neurodevelopmental phenotypes (Refs 17, 18, 22).

Experimental observations have shown that ZIKV infection significantly diminishes the overall size of brain organoids, primarily attributed to a decrease in neuroepithelium growth (Ref. 23). After infection with ZIKV, Dang et al., and Garcez et al., reported a 45.9% and 40% reduction in overall cerebral organoid size, respectively (Refs 19, 23). Additionally, Dang et al., observed a significant increase in viral copy number two days post-infection (dpi), indicating that ZIKV is a productive viral infection (Ref. 23). Furthermore, studies have shown that ZIKV infects neural progenitor cells (NPCs) and releases viral particles, as indicated by the co-localisation of ZIKVE, a marker for Zika viral envelope protein and NESTIN, a neural stem cell marker (Refs 9, 23). This infection of NPCs impairs their function, causing dysregulation of proliferation and cell cycling, reduced neurogenesis and an increase in cell death, leading to decreased organoid size (Refs 9, 23). Qian et al., showed that infection predominantly targets NPCs, while there is limited infection in immature neurons, IPCs and astrocytes in forebrain organoids (Ref. 24). This preferential infection of NPCs leads to increased NPC death, a reduction in ventricular zone (VZ) thickness, and an increase in lumen space in ventricular structures (Ref. 24). Similarly, Krenn et al., exposed organoids to ZIKV, which by 12 dpi, showed significantly smaller VZs, depleted NPC populations, and an increase in viral RNA (vRNA) expression (Ref. 10). The reduction in organoid size and increased lumen size seen in numerous studies mimic the microcephalic phenotype of ZIKV patients (Refs 9, 24). NPCs are most abundant in the first trimester, which may explain why ZIKV infection more severely affects the early stages of foetal development (Ref. 24). Further studies showed that among the proteins encoded by the ZIKV genome, NS4A and NS4B inhibit the growth of NPCs by suppressing AKT–mTOR signalling in neurospheres (Ref. 25), while NS2A impairs NPC proliferation and adherence junction formation in human forebrain organoids (Ref. 26), suggesting pathogenic mechanisms underlying ZIKV infection in NPCs.

Upon infection, the activation of cytokines known as type I interferons (IFN-I), which include multiple alpha species (IFNα) and one beta species (IFNβ), is crucial for initiating a cascade of antiviral effectors known as IFN-stimulated genes (ISGs) (Ref. 10). These ISGs play a dual role in restricting viral spread and triggering cell death (Refs 10, 23). Therefore, it has been debated whether IFN-I play a neuroprotective or detrimental role in response to ZIKV infection (Ref. 10). Exogenous administration of IFN-I, particularly IFNβ and INFα2, on ZIKV-infected organoids displayed some neuroprotective activity, as evidenced by a rescued phenotype induced by ZIKV infection (Ref. 10). IFNβ effectively inhibited ZIKV infection in organoid cultures, significantly ameliorating growth defects and reducing viral infection, demonstrating the neuroprotective role of the IFN-I system, with IFNβ showing superior efficacy compared to IFNα2 (Ref. 10). When the IFN-I immune response to ZIKV is low, greater ZIKV infection occurs due to insufficient induction of ISGs, which are crucial for neuroprotection. However, ISGs are not highly expressed by immature and progenitor cells, such as NPCs, and these cells do not rely on IFN response for antiviral defence (Ref. 10). This could offer an additional explanation why ZIKV more readily infects NPCs compared to more differentiated cells or mature neurons (Refs. 10, 18).

Dang et al., discovered that the innate immune receptor toll-like receptor 3 (TLR3) increased in expression following ZIKV infection in cerebral organoids, where they observed a decrease in overall organoid size that correlated with the kinetics of viral copy number (Ref. 23). To examine the link between TLR3 activation and disturbed neurogenesis and apoptosis, they investigated the effects of a TLR3 agonist, poly(I:C) and a TLR3 inhibitor, thiophenecarboxamidopropionate (Ref. 23). Poly(I:C) treatment led to downregulation of 41 genes including NTN1 and EPHB2, which are implicated in neurogenesis, axonogenesis, cell proliferation and apoptosis (Ref. 23). On the other hand, TLR3 inhibitor treatment rescued the phenotypic effects of ZIKV infection (Ref. 23). These results suggest a mechanistic connection between TLR3 signalling pathway and ZIKV-induced neurogenesis defects. TLR3 is highly expressed in early neurodevelopment and decreases as NPCs differentiate into mature cell lineages, potentially providing an effective therapeutic option for ZIKV infection (Ref. 23). This also offers another explanation as to why ZIKV has more severe impacts on foetal development in the first trimester (Ref. 23).

Neurospheres and brain organoids have also been used to examine how viral non-coding RNA, subgenomic flaviviral RNA (sfRNA), is involved in the death of NPCs (Refs 17, 22). It has been observed that the expression of sfRNAs from ZIKV infection leads to the downregulation of neural differentiation signalling pathways and the activation of caspase-3 and pro-apoptotic pathways (Refs 17, 22). Slonchak et al., used a placental cell line to show that sfRNAs can inhibit IFNs and therefore disrupt the innate immune response (Ref. 22). The stabilised sfRNAs, through binding to the viral protein NS5, are accumulated in infected placental cells, this accumulation inhibits STAT1 phosphorylation, thereby preventing the IFN immune response (Ref. 22). As this mechanism of ZIKV infection affects placental cells, it could be a critical aspect of how ZIKV infection is transmitted from mother to foetus, although this aspect has primarily been investigated in animal models (Ref. 22). Additionally, Slonchak et al., have shown that infection of sfRNAs-deficient ZIKV in organoids leads to less caspase-3 activation in NPCs and does not induce apoptosis or microcephaly-like phenotypes, indicating that sfRNAs are critical for viral impact (Ref. 17).

Further transcriptomic analysis found that organoids infected with wild-type ZIKV, but not with sfRNA-deficient mutant ZIKV, showed significant downregulation of genes related to signalling pathways that govern neuron differentiation and brain development such as *FOXG1* and *LHX2* (Ref. 17). Moreover, ZIKV sfRNA production during neuro-infection notably impacts the Wnt signalling pathway, which is essential for NPC differentiation (Ref. 17). The perturbation of this pathway, previously linked to ZIKV-associated microcephaly, is indicative of the involvement of sfRNA in this process (Ref. 17). These findings suggest the necessity of sfRNA for suppressing neurodevelopmental processes associated with ZIKV infection.

The AXL phosphatidylserine receptor serves as a potential entry point for ZIKV infection, facilitating viral entry into skin cells and augmenting ZIKV replication (Ref. 27). This entry pathway was demonstrated in two-dimensional (2D) fibroblast cultures, where approximately 50% of AXL-expressing cells tested positive for ZIKV infection 24 hours post-infection (hpi), and inhibiting AXL markedly decreased the number of ZIKV-positive cells (Ref. 27). Hence, to assess the association of AXL with ZIKV entry into NPCs and the potential disruption of these receptors on viral infection, brain organoids were employed. While ZIKV-infected organoids exhibited an anticipated decrease in size, AXL-knockout organoids displayed a similar reduction (Ref. 28). Moreover, the presence of caspase-3 and ZIVE was comparable in both wild-type and AXL-knockout organoids (Ref. 28). These findings suggest that AXL inhibition does not shield organoids from infection and indicate that AXL is dispensable for ZIKV infection of NPCs, diminishing its viability as a therapeutic target (Ref. 28). This highlights the importance of 3D model systems in addition to 2D cultures (Ref. 28).

As ZIKV infection preferentially targets NPCs, it is critical to find ways to specifically treat the NPCs. Thus, brain organoids have been utilised for the identification and validation of therapeutic drugs for ZIKV. Brain organoids were treated with sofosbuvir (SOF), a clinically approved drug for hepatitis C virus (HCV), inhibited ZIKV replication by targeting its RNA polymerase, a conserved protein among Flaviviridae family members, and by enhancing A-to-G mutations (Refs 29, 30). Alternatively, RNA interference (RNAi) is a mechanism that is a part of the innate antiviral immune response, producing virus-derived small interfering RNAs (vsiRNA) (Ref. 18). ZIKV infection induces a significant production of vsiRNAs specifically in NPCs by efficiently processing vRNA to vsiRNA through the RNAi machinery, which is not observed in more differentiated, postmitotic cells (Ref. 18). Xu et al., demonstrated that the removal of key RNAi machinery components notably increased ZIKV replication in NPCs, underscoring the critical antiviral role of RNAi during ZIKV infection in these cells (Ref. 18). Moreover, enoxacin, a broad-spectrum antibiotic known for its RNAi-enhancing properties, exhibited potent anti-ZIKV activity in NPCs and other RNAi-competent cells (Ref. 18). Notably, treatment with enoxacin completely prevented ZIKV infection and mitigated ZIKV-induced microcephalic phenotypes in brain organoids (Ref. 18).

### Rubella virus

The Rubella virus (RV), an enveloped, single-stranded RNA virus belonging to the Matonaviridae family, with humans being RV’s only natural host (Ref. 31). Despite advancements in vaccination efforts achieving global coverage of approximately 69%, RV endemics persist within Africa, the Eastern Mediterranean, and South-East Asia (Refs 31, 32). RV’s impact on pregnancy is profound, as it can cause a spectrum of congenital defects known as Congenital Rubella Syndrome (CRS) (Refs 31, 33). CRS manifestations range from severe developmental disorders, including microcephaly, ASD, schizophrenia, deafness, and cardiac anomalies to miscarriages and stillbirths (Refs 31, 33). The transplacental route of infection is evidenced by the presence of RV in the blood and placenta of infected foetuses (Refs 31, 34). With the World Health Organization reporting up to a 90% chance of vertical transmission in cases of maternal infection, an increased understanding of this process is paramount (Ref. 32). One clinical study reported 69% of patients with CRS, that survived to 18 months, had some form of neurological disability, including ASD, seizures and motor defects (Ref. 35). Studies have suggested that the foetus is most vulnerable to RV infection, and associated developmental risks, prior to gestational week 16 (GW 16) (Refs 32, 33). It is established that in the first 6 GWs, the foetus is unable to produce its own antibodies against the RV (Ref. 34). After 6 GWs, maternal rubella-specific antibodies can be detected in the foetus, but these are at levels insufficient to protect the foetus from damage (Ref. 34). However, after GW 16, the foetuses own immune response, in addition to maternal antibody transfer, is enough to protect the foetus from damage (Ref. 34).

Although the mechanisms behind foetal RV infection, maternal vertical transmission to the brain and the resulting pathology of CRS are still poorly understood, many mechanisms have been suggested (Ref. 31). Popova et al., used cerebral organoids co-cultured with mid-gestation primary human microglia, to delineate RVs cellular targets within the brain (Ref. 31). Interestingly, microglia in monoculture showed low levels of RV infection but when co-cultured with neurons, glial cells and NPCs, the infection rate increased from 2% to 60% (Ref. 31). This was similarly shown in the 3D organoids, where the co-cultured organoids showed microglia infection and organoids without the engraftment of microglia showed minimal infection (Ref. 31). RV infection resulted in an increased IFN response in organoids, including *IFI27, IFI6* and *IFITM3;* however, this response was less significant with the engraftment of microglia cells, except for IFITM3, which was highly upregulated with the presence of microglia (Ref. 31). Utilising single-cell RNA sequencing (scRNA-seq), RV infection was also shown to initiate an IFN response in neurons and NPCs (Ref. 31). However, the effect of RV infection on microglia could not be confirmed through scRNA-seq, as the canonical microglia marker P2RY12 was not detected in the cell populations, the authors attributed this to a loss of cells during cell dissociation and the small starting population (Ref. 31). Additionally, scRNA-seq revealed that the *NOVA1*, which regulates alternative splicing in the central nervous system (CNS) and is linked to neurological diseases, was altered by both the presence of microglia and RV infection (Ref. 31). This was confirmed through immunohistochemistry staining showing that infection of microglia with RV decreased the number of NOVA1^+^ IPCs (Ref. 31). Furthermore, *NFIB* and *NFIA*, genes associated with gliogenesis in embryonic brain development, were specifically downregulated in RV-infected organoids without microglia (Ref. 31). The disruption of these genes in early development is associated with neurodevelopmental defects and intellectual disability (Ref. 31). Due to the human-specific nature of RV, further human brain organoid studies, expanding on the work produced by Popova et al. in 2023, would help increase the understanding of CRS mechanisms.

### Human cytomegalovirus

HCMV is a betaherpesvirus, resulting in lifelong infection and is a leading cause of neurodevelopmental defects, such as microcephaly, intellectual disability, cerebral palsy and seizures (Refs 36, 37, 38). There are three stages of infection: (1) the ‘immediate early’ stage which involves viral DNA synthesis and replication, along with inhibition of innate immune response (Ref. 36); (2) the ‘early stage’ of viral genome replication and packaging (Ref. 36) and (3) the ‘late stage’ expression of structural genes and proteins (Ref. 36). The virus can be active, causing an immune response or remain latent in hematopoietic progenitors and monocytes with no replication (Ref. 36). Given that the virus can remain dormant, primary maternal infection or secondary maternal viral reactivation can occur during pregnancy, allowing for transmission from mother to foetus (Refs 38, 39).

Congenital HCMV infection and its neuropathogenesis are still poorly understood (Refs 38, 40). HCMV infection, progression and mechanisms are species-specific, which makes the use of animal models difficult, although some research has been done using rodent and rhesus monkey models (Refs 37, 38). Therefore, a humanised model, such as human organoids, that can accurately recapitulate the disease is critical (Refs 37, 38). Cerebral organoids grown from HCMV-infected iPSCs displayed a reduction in organoid size and structures, large vacuoles and cyst formation, as well as necrosis, resembling the microcephalic phenotype clinically observed (Ref. 40). HCMV infection also resulted in disrupted NPC differentiation and function, cell necrosis, inflammation, an increase in infiltrating macrophages and activated microglia (Refs 36, 38). In a study conducted by O’Brien et al., downregulation of neurodevelopmental genes including *NES, SOX2/4, FOXG1, DMRTA2* and *EMX1* was observed (Ref. 36). This led to the disruption of multiple signalling pathways, including those involved in cell signalling and differentiation, affecting not only cells with high viral gene expression but also a broader range of cells (Ref. 36). Viral proteins IE1 and IE2 were detected in HCMV-infected organoids, alongside disrupted signalling pathways. However, O’Brien et al., demonstrated that reducing the levels of IE1 and IE2 proteins in the infected organoids was not sufficient to rescue the neurodevelopmental networks, indicating that there are other dominant mechanisms for HCMV infections (Ref. 36). While IE1 and IE2 are necessary for lytic infection and reactivation from viral latency, solely targeting these proteins to limit viral replication and gene expression may not alleviate the widespread neurodevelopmental impacts induced by HCMV infection (Refs 36, 41).

Currently, there are no approved treatments for HCMV infection during pregnancy (Refs 36, 37, 40). However, in symptomatic infants, children and adults, HCMV is managed by antiviral drugs, including (val)ganciclovir, cidofovir, foscarnet and letermovir which inhibit viral DNA synthesis or target viral DNA packaging (Ref. 37). However, antiviral resistance occurs with all the currently used compounds (Ref. 37). Sison et al., have trialled the use of maribavir (MBV) in HCMV-infected cortical organoids, which showed loss of NPC rosette structures and expression patterns in SOX2^+^ and PAX6^+^ cells and a lack of CTIP2^+^ cells (Ref. 37). MBV treatment was able to restore the rosette structure and CTIP2^+^ cells, suggesting that MBV restores the function of NPCs and their ability to differentiate into neurons (Ref. 37). Sison et al., also performed calcium imaging on neurons and astrocytes, generated from HCMV-infected dissociated organoids and showed that while these cells were electrophysiologically active, the HCMV-infected organoids had a lower baseline of calcium activity and reduced response to ATP stimulation, disrupting the normal ion response that is essential for neurodevelopment (Ref. 37). Treatment with MBV was able to increase the number of uninfected cells responding to ATP and potassium chloride stimulation but had a limited effect on the HCMV-infected cells (Ref. 37). This study showed that MBV was able to reduce the spread of HCMV infection and rescue some phenotypic changes but failed to restore function in HCMV-infected neurons (Ref. 37). Therefore, in combination with other neuroprotective agents, MBV could help to reduce the developmental defects caused by HCMV (Ref. 37).

To model potential therapeutic targets, Sun et al., used two strains of HCMV, TB40/E, a clinical-like strain and Towne, an attenuated laboratory strain, to infect brain organoids (Ref. 38). TB40/E expresses an envelope pentamer complex (PC) and was able to efficiently infect and propagate in brain organoids, which resulted in a microcephaly-like phenotype (Ref. 38). In contrast, the Towne strain does not express PC and could not efficiently infect the brain organoids, thus having no impact on the organoid size, indicating that PC is critical for viral infection and the induction of microcephaly-like phenotypes (Ref. 38). TB40/E was able to infect and disrupt SOX2^+^ progenitor cells at the core and neurons in TUJ1^+^ neuronal layer of the organoids (Ref. 38). Infected organoids also showed an increase in apoptosis and a decrease in proliferation, indicated by BrdU and caspase-3 staining (Ref. 38). RNA sequencing revealed that TB40/E infection resulted in the downregulation of calcium signalling-related genes, including *ENO2, BNIP3* and *PDK* (Ref. 38). This was followed by gene ontology analysis which revealed that genes significantly downregulated in TB40/E-infected organoids are involved in neurodevelopment, including astrocyte development and pathways involved in calcium signalling (Ref. 38). Conversely, genes involved in immune and inflammatory responses were upregulated in TB40/E-infected organoids (Ref. 38). Additionally, Sun et al., demonstrated that PDGFRα and EGFR cellular receptors are required for viral entry, as the overexpression of PDGFRα and EGFR in NPCs increased cell susceptibility to HCMV infection, specifically EGFR for PC-mediated entry (Ref. 38). To treat these impacts of HCMV infection, Sun et al., employed neutralising antibodies (NAbs), which have previously been used to interfere with the viral infection (Ref. 38). When organoids were treated with the anti-HCMV PC NAbs, 1B2 and 62–11, a lower rate of infection was observed along with normal organoid growth (Ref. 38). This NAb-rescued phenotype showed improvements in many clinical symptoms including microcephaly-like phenotypes and normalised calcium-signalling, indicating that NAbs can be an effective therapeutic against HCMV infection (Ref. 38). Current research indicates that PC-specific NAbs can be transferred from mother to foetus and prevent severe neurodevelopmental malformations, suggesting that vaccine-induced or passively administered NAbs could reduce the impact of vertical transmission and the impact of HCMV infection in foetal development (Ref. 38).

### Herpes simplex virus

HSV is in the Herpesviridae family, with HSV-1 being the most common, orally transmitted virus and HSV-2 being a sexually transmitted infection causing genital herpes (Ref. 42). HSV presents a significant risk to developing foetuses as it is the second most prevalent TORCH pathogen and can be transmitted across the placental barrier. Without treatment, the infected foetus has a 60% mortality rate (Refs 42, 43). HSV is another lifelong infection that has been linked to neurodevelopmental disorders including attention deficit hyperactivity disorder (ADHD), ASD, intellectual and learning disabilities and cerebral palsy (Refs 42, 43). Furthermore, HSV can lead to herpes simplex encephalitis (HSE), an often-fatal disease of the CNS, characterised by neuroinflammation (Refs 42, 43, 44). Brain organoids exposed to HSV-1 experienced a loss of tissue integrity and impaired growth with fewer and smaller VZs, linking HSV-1 infection of early-stage organoids to microcephaly (Ref. 10).

Upon HSV-1 infection in cerebral organoids, Qiao et al. observed impairment of neural differentiation, dysregulated neurogenesis and disruption of cortical layers (Ref. 42). Specifically, HSV-1 infection was observed to decrease the expression of MAP2 and TUJ-1 mRNA, accounting for the inhibition and disruption of neural differentiation processes (Ref. 42). Additionally, HSV-1 infection resulted in an increase in astrocyte activation and an increase in microglia proliferation and active CD11b expression, as indicated by the increase in IBA1^+^ and CD11b^+^ cells (Ref. 42). This increase in microglia is associated with an increase in pro-inflammatory cytokines, TNF-α and IL-6, resulting in high levels of neuroinflammation (Ref. 42). It is important to note that the authors claim of increased microglia proliferation upon HSV-1 infections is based on the observed increased expression of IBA1 and CD11b proteins. However, there is no data to confirm that these IBA1^+^ and CD11b^+^ cells are mature microglia, as microglia do not inherently populate cerebral organoids. It is possible that these cells may be more general macrophage/myeloid cells. Further validation, which could include co-culturing cerebral organoids with microglia, is required to substantiate these effects of HSV-1 infection on microglia.

Krenn et al., investigated the effects of INFs on HSV-1-infected organoids (Ref. 10). It was observed that IFNα2 treatment was sufficient to rescue organoid architecture and growth defects, while also reducing HSV-1 infection by suppressing HSV-1 transcription (Ref. 10). Alternatively, acyclovir (ACV), a potential therapeutic antiviral drug, has been used to effectively reduce the spread of HSV-1 infection in cerebral organoids (Ref. 43). However, while ACV allows for continued differentiation of early organoids, the ACV-treated, HSV-1-infected organoids were still smaller than the control infected organoids at 15 dpi and tissue degradation was still observed (Ref. 43). This inability of ACV to rescue the diseased HSV-1 phenotype is believed to be due to ACV resistance that emerges by 15 dpi in brain organoids, validated by ACV-resistant particles found in the culture medium (Ref. 43). This resistance is aggravated by the infection of NPCs, resulting in continued neurodevelopmental abnormalities even after treatment with ACV (Ref. 43). The treatment of HSE using ACV was modelled in brain organoids and analysed through high-throughput scRNA-seq (Ref. 44). Upon HSV-1 infection, there was a significant increase in TNF signalling, contributing to the high levels of clinical neuroinflammation seen (Ref. 44). With ACV treatment, the replication of the virus was stopped but the neuroinflammation, and therefore neurological disorders, persisted (Ref. 44). To combat this, a combinatorial anti-viral/anti-inflammatory treatment of drugs such as necrostatin-1 or bardoxolone methyl with ACV was trialled and found to reduce immune activation, reducing the damage to neuronal cells and preserving neuroepithelial integrity (Ref. 44). This underscores the importance of targeting both the viral infection and resulting inflammation when developing an effective therapy for HSV-1.

## SARS-CoV-2/COVID-19

Declared a pandemic by the World Health Organization on 11 March 2020, Coronavirus disease 2019 (COVID-19), caused by SARS-CoV-2, rapidly emerged as a global health crisis (Refs 45, 46). By the end of 2020, the global death toll was estimated to be 3 million, escalating to approximately 775 million cases as of March 2024 (Refs 45, 46). As a member of the *Coronaviridae* family, SARS-CoV-2 is characterised by its enveloped structure and positive-sense single-stranded RNA (Ref. 47). Although primarily recognised as a respiratory disease, SARS-CoV-2 exhibits wide-ranging effects on multiple organ systems, including the brain, heart, liver, kidneys and gastrointestinal tract (Refs 48, 49, 50). SARS-CoV-2 infection has been associated with a wide variety of neurological symptoms including headaches, strokes, seizures, encephalitis, neurodegeneration and psychosis, highlighting the impact viral infection has on the brain (Refs 51, 52). Vertical transmission of SARS-CoV-2 has been well established through the detection of SARS-CoV-2 in the placenta, amniotic membranes, amniotic fluid and potentially in the cord blood of neonates from infected mothers (Refs 53, 54, 55). This elucidates the potential impact that SARS-CoV-2 has on the developing foetus, influencing foetal brain development and thereby increasing the risk of neurodevelopmental disorders (Ref. 54). Furthermore, there is an indication that COVID-19 could be considered a congenital disease, with the possibility of foetal neuroinvasion and active infection occurring primarily during the second and third trimesters (Refs 49, 54, 56). The implications of vertical transmission for COVID-19 on neurodevelopment remain largely unexplored. However, preliminary studies indicate a possible association with MIA, due to viral infection and a range of neurodevelopmental conditions, such as ASD, ADHD, schizophrenia and anxiety (Refs 57, 58).

SARS-CoV-2 enters the host cells primarily through binding the angiotensin-converting enzyme 2 (ACE2), followed by the cleavage of its Spike protein by transmembrane serine protease 2 (TMPRSS2) or FURIN, facilitating entry and triggering an inflammatory response (Refs 16, 47, 49). ACE2 expression in the brain, particularly within the choroid plexus (ChP), is significant; however, neurons and other brain cell types exhibit relatively low ACE2 levels (Refs 49, 50). This discrepancy hints at the potential role of alternative receptors such as neuropilin 1 (NRP1), which is present in neurons and astrocytes, in mediating the virus’s entry into the CNS, suggesting a multifaceted mechanism for SARS-CoV-2 neuroinvasion (Ref. 53). Organoid studies have demonstrated SARS-CoV-2’s capability to infect mature neurons, demonstrated by the co-localisation of MAP2^+^ and TUJ-1^+^ cells with viral components such as the nucleocapsid protein (NP), Spike protein and viral RNA (Refs 50, 59, 60).

The ChP, a layer of epithelial cells within the brain, plays a critical role in protecting the brain from pathogens through its interactions with the blood–brain barrier (BBB) and the blood–cerebrospinal fluid barrier (B-CSF-B) (Refs 5, 61) (Figure 2). It is instrumental in the brains immune and inflammatory response by secreting proinflammatory cytokines and facilitating immune cell interactions (Refs 5, 61, 62). Notably, the ChP emerges as the brain region most susceptible to infection, potentially elucidating the neuropathological effects induced by the virus (Refs 5, 52, 55). This observation emphasises the importance of the ChP not only as a critical site of viral entry into the CNS but also as a possible focal point for understanding the virus’s impact on neurological health.

ChP organoids, expressing OTX2, AQP1 and TTR, have been used to demonstrate that SARS-CoV-2 can infect ChP cells effectively (Refs 5, 52). This viral entry was shown to disrupt the integrity of tight junctions within the ChP, compromising the B-CSF-B and leading to structural breakdown in the organoids (Ref. 5). Such disturbances enable the infiltration of pathogens, immune cells and proinflammatory cytokines into both the brain organoids and the CSF, providing insight into the potential neuropathological impacts of the virus (Ref. 5). Notably, a significant upregulation of ACE2 and TMPRSS2 expression was observed in mature, lipoprotein-producing ChP cells, particularly abundant during later developmental stages (Refs 5, 63). This susceptibility suggests that the ChP, especially its mature cell populations, may serve as a pivotal, alternate entry point for the virus into the CNS, potentially culminating in the neuroinflammation noted in COVID-19 patients (Ref. 5).

Although the ChP appears to be most highly sensitive to SARS-CoV-2 infection, studies have shown that other brain cells can be infected (Refs 47, 51). Tiwari et al., used brain organoids to show that SARS-CoV-2 infection of neurons leads to activation of the complement system and immune response, shown by TLR3/7 and OAS2 expression (Ref. 47). This infection is further characterised by an upregulation of apoptotic genes and necrosis pathways, alongside alterations in entry factors such as PLASMIN and NRP1, and a concurrent downregulation of anti-apoptotic genes, including *BCL2* and *BAX* (Ref. 47). Contrarily, McMahon et al., report that while SARS-CoV-2 established a non-productive infection in neurons and NPCs, indicating limited viral replication, a productive infection was achieved within the ChP and astrocytes (Ref. 51). This is supported by the co-localisation of viral NP with markers such as 5-HT2C for the ChP, GFAP for astrocytes and NESTIN for RGCs, underscoring the susceptibility of these cell types to infection (Ref. 51). The differences between studies in cell susceptibility to SARS-CoV-2 infection highlight how the exact mechanisms behind the viral pathogenesis in the brain are still up for debate (Ref. 16).

The critical role of brain barriers in viral infections has brought pericytes, key regulators of the BBB, neurogenesis and neuroinflammation, into focus alongside astrocytes for their contributions to BBB maintenance (Refs 51, 64). Studies involving pericyte-containing cortical organoids (PCCOs) have revealed that these organoids are characterised by the expression of mature astrocytic markers and the accumulation of laminin-β at the basement membrane, alongside a marked shift towards differentiated neuronal populations (Ref. 64). These organoids, which notably harbour fewer progenitor cells, as indicated by the elevated expression of astrocytic and neuronal differentiation markers such as GFAP, TBR1, DCX and STMN2, demonstrate a susceptibility to SARS-CoV-2 infection (Ref. 64). Remarkably, PCCOs exhibit up to a 50-fold increase in SARS-CoV-2 infection rates compared to organoids without pericytes, with viral NP detected in both astrocytes and pericytes (Ref. 64). This significant finding suggests that pericytes within PCCOs act as central hubs for viral replication, resulting in viral spread to adjacent astrocytes, subsequently triggering an IFN-1 immune response (Ref. 64). This mechanism mirrors clinical observations in COVID-19 patients, who exhibit neurological symptoms such as strokes, haemorrhages, seizures and encephalitis, among others (Refs 55, 64). Thus, pericytes play a pivotal role in the neuropathological effects of SARS-CoV-2, offering a novel insight into the viral mechanisms of CNS invasion and highlighting potential therapeutic targets for mitigating its neurological impact.

While ACE2 expression in the brain has shown to be low compared to lung tissues, it is still a critical entry factor for SARS-CoV-2 infection (Refs 47, 60). ACE2 has been detected in the ChP and may be present on the surface of other cell types, promoting entry into cells (Refs 5, 52, 60). Therefore, inhibiting ACE2 is a potential for therapeutics. To investigate this, Song et al., pre-treated brain organoids with anti-ACE2 antibodies and showed significant inhibition of SARS-CoV-2 infection (Ref. 60). Alternatively, patient-derived CSF, which contained antibodies against SARS-CoV-2-specific Spike proteins, was used to treat organoids and effectively prevented infection (Ref. 60). Mesci et al., have shown that in 8-week-old cortical organoids, SARS-CoV-2 infection peaked 48 hpi (Ref. 53). Using these organoids, they were able to trial an approved drug, SOF, which has previously been used to effectively block vertical transmission of Hepatitis C (HVC) and ZIKV (Refs 30, 53). SOF was found to be effective at reducing SARS-CoV-2 vRNA, with 20 μM treatment having the highest inhibition without any cell death and was able to rescue the disease phenotypes (Ref. 53). Thus, indicating that SOF holds the potential to be an effective therapeutic to block vertical transmission of SARS-CoV-2 (Ref. 53).

Investigating the neurotropism of SARS-CoV-2, Wang et al., found that 60-day-old organoids, when exposed to the virus, showed notable infection in neurons (Ref. 59). This was demonstrated by the co-localisation of the Spike protein and TUJ-1^+^cells, indicative of neural infection, and an increased presence of viral NP (Ref. 59). Significantly, organoids and 2D cultures rich in astrocytes presented a substantially higher rate of neuronal infection compared to neuron-only cultures, emphasising the critical role of astrocytes in potentially facilitating SARS-CoV-2’s neurological invasion (Ref. 59). Interestingly, treatment with anti-viral drug remdesivir, in 2D cultures not only reduced Spike-positive neurons and astrocytes but also mitigated disease phenotypes such as nuclear fragmentation and neurite length reduction, pointing to its potential therapeutic efficacy against SARS-CoV-2’s neuroinvasive properties (Ref. 59).

SARS-CoV-2’s ability to invade the brain, induce neural cell death and its detection in the placenta of exposed pregnant women stresses the urgent need to unravel the mechanisms of vertical transmission and potential neurodevelopmental consequences for developing foetuses (Ref. 54). Consequently, advancing our understanding of SARS-CoV-2’s impact on foetal development is imperative, not only to ascertain its classification as a congenital disease but also to evaluate its candidacy as a TORCH pathogen (Refs 13, 54). Addressing these research gaps is essential for developing preventative and therapeutic strategies to protect the most vulnerable from the long-term neurological effects of COVID-19 (Ref. 13).

## Limitations and future directions

Human brain organoids provide a suitable platform for exploring a range of pathogens and their associated risk for neurodevelopmental disorders; however, they do have several limitations. The absence of functional vasculature in organoids limits their ability to efficiently exchange nutrients and gases, which in turn restricts their growth (Ref. 1). As a result, organoids remain significantly smaller than human organs, typically reaching a maximum size of about 4 mm in diameter (Ref. 1). These conditions can further limit long-term studies needed to track the developmental trajectory of infected neonates. Furthermore, brain organoids lack key interactions with critical immune components, such as microglia, the choroid plexus and the meninges, limiting their ability to fully replicate the immune response to viral exposure (Refs 44, 50). Recently, substantial progress has been made in developing brain organoids that incorporate microglia. Two main approaches have emerged: adjusting culture conditions to support the endogenous development of microglia within brain organoids and integrating iPSC-derived microglia into organoid systems. However, functional validation of these microglia remains crucial to ensure they faithfully replicate the characteristics and functions of microglia in the human foetal brain (Refs 65, 66, 67, 68, 69). Additionally, brain organoids lack the BBB, B-CSF-B and placental/maternal interactions, which have been highlighted as critical components of congenital defects and neurodevelopment, thereby limiting the early-stage organoids ability to replicate placental interactions (Ref. 7). Without incorporating these maternal factors into the brain model system, the exact mechanisms of vertical transmission, and the influence of MIA, will remain poorly understood. Finally, determining an appropriate viral titer *in vitro* is challenging, as viral exposure in organoids does not mimic the complex infection barriers present *in* *vivo*.

## Conclusion

The development and application of 3D brain organoids represent a significant technological advancement with substantial potential, as they can model both early and late stages of neurodevelopment and provide valuable insights into neurodevelopmental disorders. Brain organoids have proven effective in modelling congenital diseases and can be exposed to many environmental factors and genetic manipulations, thereby reducing the need for primary tissue and dependence on animal models. Their extensive use in studying TORCH pathogens has aided the discovery of mechanisms of infection, drug screening and the development of treatments (Table 1). Ongoing advancements in integrating key components such as microglia, vasculature and the choroid plexus into brain organoids, alongside innovations in bioengineering and organoid maturation techniques, will significantly enhance their capacity to more faithfully replicate human brain development and pathology. These improvements are particularly critical for studying viral infections, allowing for more accurate modelling of disease mechanisms, immune responses and the potential development of therapeutic interventions.
